# Evaluating surgical techniques for incarcerated incisional hernia: laparoscopic vs. Open repair in a tertiary care setting

**DOI:** 10.1007/s10029-025-03311-0

**Published:** 2025-03-12

**Authors:** Kayhan Özdemir, Emrah Akin, Ali Muhtaroğlu, Burak Kamburoğlu, Emre Gönüllü, Zülfü Bayhan, Fatih Altintoprak

**Affiliations:** 1 Department of General Surgery, Sakarya University Faculty of Medicine, Sakarya, Turkey; 2 Department of General Surgery, Giresun University Faculty of Medicine, Giresun University Training and Research Hospital, Aksu District, Mehmet İzmen Street, Number:145, Giresun, 28100 PC Turkey; 3 Department of General Surgery, Derince Training and Research Hospital, Kocaeli, Turkey; 4 Department of General Surgery, Division of Gastroenterological Surgery, Giresun University Faculty of Medicine, Sakarya, Turkey

**Keywords:** Incarcerated incisional hernia, Laparoscopic repair, Emergency surgery, Hernia repair techniques, Perioperative outcomes

## Abstract

**Purpose:**

This study aims to compare the outcomes of laparoscopic versus open repair techniques in patients undergoing emergency surgery for incarcerated incisional hernia in a tertiary care setting.

**Methods:**

A prospective evaluation was conducted on 45 patients who underwent emergency laparoscopic and open repair for incarcerated incisional hernia between 2018 and August 2021. Patients were divided into two groups based on the surgical technique used: laparoscopic (*n* = 15) and open repair (*n* = 30). Key variables analysed included demographic data, body mass index, American Society of Anesthesiologists scores, operative time, perioperative bleeding, length of hospital stay, postoperative complications, European Hernia Society Quality of Life pain score, and recurrence rates during follow-up.

**Results:**

Significant differences were found between the laparoscopic and open repair groups regarding pain scores, length of hospital stay, and amount of perioperative bleeding. The laparoscopic repair group demonstrated reduced pain, shorter hospital stays, and less perioperative bleeding compared to the open repair group.

**Conclusion:**

This study shows that laparoscopic repair for incarcerated incisional hernia offers significant advantages over open repair. These findings support the preference for laparoscopic repair in the emergency surgical management of incarcerated incisional hernia in appropriate patients.

**Supplementary Information:**

The online version contains supplementary material available at 10.1007/s10029-025-03311-0.

## Introduction

Incarcerated incisional hernias represent a significant clinical challenge, often necessitating emergent surgical intervention [1]. Incisional hernias occur in approximately 10–20% of patients who have undergone abdominal surgery, with the risk of incarceration estimated to be around 6–15% over a patient’s lifetime [2]. This condition arises when part of the intestine becomes trapped within the hernia sac, leading to bowel obstruction, ischemia, and potential perforation. The pathophysiology involves the protrusion of abdominal contents through a weakened area in the abdominal wall, typically at the site of a previous surgical incision [3].

Clinically, incarcerated incisional hernias present with acute abdominal pain, vomiting, and signs of bowel obstruction such as distension and the inability to pass gas or stool. Physical examination often reveals a tender, irreducible mass at the hernia site. Radiological imaging, particularly computed tomography (CT) scans, is instrumental in diagnosing and assessing the extent of bowel involvement, which is crucial for surgical planning [4].

The management of incarcerated incisional hernias is critical, given the potential for severe complications such as bowel strangulation and necrosis, which can significantly increase morbidity and mortality rates [5]. The necessity of bowel resection depends on the viability of the trapped intestine, which must be assessed intraoperatively. Prompt surgical intervention is essential to prevent irreversible ischemic damage to the bowel [6].

Traditionally, open surgical repair has been the standard approach for the treatment of incarcerated incisional hernias. This technique typically involves a large abdominal incision to access the hernia sac, reduce the incarcerated contents, and repair the defect, often with the placement of a mesh to reinforce the abdominal wall [7]. While effective, open repair is associated with significant perioperative morbidity. Patients undergoing open surgery often experience increased pain due to the larger incision, prolonged hospital stays for recovery, and higher rates of perioperative bleeding and wound complications [8]. Additionally, the risk of surgical site infections and longer-term complications, such as hernia recurrence, remains a concern with open repair [9].

With advances in minimally invasive surgery, laparoscopic repair has emerged as a viable alternative [10]. This approach involves several small incisions through which a camera and surgical instruments are inserted. The laparoscopic technique offers the potential for reduced postoperative pain, shorter hospital stays, decreased perioperative blood loss, and faster recovery times. Furthermore, laparoscopic surgery provides better visualisation of the abdominal cavity, which can facilitate more precise and effective hernia repair [11]. Studies have shown that laparoscopic repair is associated with lower rates of wound infections and fewer postoperative complications than open repair [12].

Incarcerated incisional hernias most commonly develop after surgeries involving midline incisions, such as those performed for colorectal, gynaecological, and upper gastrointestinal procedures [13]. Factors that contribute to the development of incisional hernias include wound infection, obesity, smoking, and poor nutritional status. The healing process of the abdominal wall is often compromised in these patients, leading to a weakened area susceptible to herniation [14].

The European Hernia Society and the American Hernia Society emphasise the need for robust comparative studies to guide clinical decision-making. Given the potential advantages of laparoscopic repair, such as enhanced recovery profiles and lower complication rates, understanding its role in the emergency context is crucial for optimising patient outcomes.

This study aims to fill the existing knowledge gap by providing a prospective evaluation of laparoscopic and open repair techniques in patients undergoing emergency surgery for incarcerated incisional hernia in a tertiary care health institution.

In our study, we aimed to determine the most effective and safe surgical approach for this high-risk patient population by analysing key variables such as operative time, perioperative bleeding, length of hospital stay, postoperative complications and quality of life measures.

## Methods

This retrospective study was approved by the local ethics committee of Sakarya University Faculty of Medicine (Approval Number: 71522473/050.01.04/32180-297). The research was conducted following our institution’s ethical standards and the 1964 Helsinki Declaration and its later amendments. Due to the retrospective nature of this study, the requirement for informed patient consent was waived. The study was executed at Sakarya Training and Research Hospital, located in Sakarya province, utilising a retrospective cohort design.

This prospective study evaluated 45 patients who underwent emergency surgery for incarcerated incisional hernia between 2018 and August 2021. In a tertiary care hospital, the patients were divided into two groups based on the surgical technique employed: laparoscopic repair and open repair.

Patients were included in the study if they met the following criteria: aged 18 years and older, diagnosed with incarcerated incisional hernia requiring emergency surgical intervention, underwent laparoscopic repair with intraperitoneal onlay mesh (IPOM) technique and fascial closure, or underwent open repair with inlay mesh technique. Additionally, patients had to present clinical and radiological findings suggestive of small bowel obstruction. Patients were excluded if they required resection for strangulated hernia, had previous hernia repair using mesh, had comorbid conditions that precluded general anaesthesia, had incomplete medical records or follow-up data, or declined to participate in the study.

The laparoscopic repair involved the IPOM technique with fascial closure, taking into account clinical and radiological findings of small bowel obstruction. The open repair was performed using the inlay mesh technique.

For each patient, the following data were collected: demographic data (age, gender), body mass index (BMI), American Society of Anesthesiologists (ASA) scores, operative times, perioperative bleeding, postoperative hospitalisation times, European Hernia Society Quality of Life (EuraHS QoL) pain score (Table 1), perioperative and postoperative complications, and recurrence during follow-up.

**Table 1:** European hernia society quality of life pain score (Postoperative) [15]

Pain at the side of the hernia repair												
In rest (lying down)	0	1	2	3	4	5	6	7	8	9	10	
During activities (walking, biking, sports)	0	1	2	3	4	5	6	7	8	9	10	
Worst pain felt during the last week	0	1	2	3	4	5	6	7	8	9	10	
***0 = no pain 10 = worst pain imaginable***												
**Restrictions of activities because of pain or discomfort at the side of the hernia repair**												
Daily activities (inside the house)	0	1	2	3	4	5	6	7	8	9	10	X
Outside the house (walking, biking, driving)	0	1	2	3	4	5	6	7	8	9	10	X
During sports	0	1	2	3	4	5	6	7	8	9	10	X
During heavy labour	0	1	2	3	4	5	6	7	8	9	10	X
***0 = no restriction 10 = completely restricted X = if you do not perform this activity***												
**Cosmetic discomfort**												
The shape of your abdomen	0	1	2	3	4	5	6	7	8	9	10	
The site of the hernia and the scars	0	1	2	3	4	5	6	7	8	9	10	
***0 = very beautiful 10 = extremely ugly***												

### Statistical analysis

Data was analysed using the Statistical Package for the Social Sciences (SPSS) 26.0 Statistics package program. The suitability of the numerical variables of the patients to the normal distribution was determined by Kolmogorov-Smirnov test. The chi-square test compared patients’ descriptive features and pathology findings. The Independent Sample T Test or Mann-Whitney U test was used to compare patients’ age and laboratory parameters. The significance level was carried out in the study by considering the values of 0.05.

## Results

The study included 45 patients who underwent emergency surgery for incarcerated incisional hernia. The patients were divided into two groups: open repair (*n* = 30) and laparoscopic repair (*n* = 15).

### Open repair group

The mean age of the patients in the open repair group was 64.8 years (range 51–87), with 30% being female (*n* = 9) and 70% male (*n* = 21). The mean BMI was 25.8 (range 21.4–28). Regarding the ASA scores, 10 patients (33.3%) were ASA 2, and 20 patients (66.6%) were ASA 3. The mean operation time for this group was 102.8 min (range 75–150 min). The mean perioperative bleeding was 276.6 cc (range 100–400 cc). The mean hospital stay was 3.6 (range 2–8 days). The mean EuraHS QoL pain score was 51.1 (range 20–70). One patient experienced a small bowel injury during the passage of abdominal layers, which was managed with primary repair. During a mean follow-up period of 12.8 months (range 1–23 months), recurrence occurred in one patient who had a previous diagnosis of chronic obstructive pulmonary disease.

### Laparoscopic repair group

The mean age of patients in the laparoscopic repair group was 51.3 years (range 37–67), with 40% being female (*n* = 6) and 60% male (*n* = 9). The mean BMI was 29 (range 22.3–32.6). In terms of ASA scores, 20% of patients (*n* = 3) were ASA 1, 40% (*n* = 6) were ASA 2, and 40% (*n* = 6) were ASA 3. The mean operation time was 93.3 min (range 75–120 min). The mean amount of perioperative bleeding was 81.6 cc (range 50–250 cc). The mean hospital stay was 1.8 days (range 1–3 days). The mean EuraHS QoL pain score was 17 (range 10–40). No postoperative complications were observed in this group. During a mean follow-up period of 11.4 months (range 3–24 months), recurrence was noted in one patient who was a farmer.

### Comparative analysis

Significant differences were observed between the two groups regarding ASA scores, pain scores, length of hospital stay, and amount of perioperative bleeding (*p* < 0.05). The laparoscopic repair group demonstrated lower ASA scores, reduced pain, shorter hospital stays, and less perioperative bleeding compared to the open repair group.

A comparison of the groups according to demographic characteristics is presented in Table 2. The other variables compared are shown in Table 3.

**Table 2:** Comparison of demographic characteristics of patients between two groups

Patients’ characteristics		Open group (*n*:30)		Laparoscopic group (*n*:15)		*p*
		Number	%	Number	%	
Gender	Female	9	30	6	40	0.186
	Male	21	70	9	60	
		Mean ± SD		Mean ± SD		
BMI^t^		25.8 ± 2.8		29.0 ± 3.2		0.47
Age^t^		64.8 ± 8.0		51.3 ± 8.5		0.09

*p < 0,05, **p < 0,01, 𝜒2:Chi-square test (Categorical data), t:independent sample T test, SD: Standart deviation, min: minimum, Max: maximum

**Table 3:** Comparison of variables between two groups

Variables			Open group (*n*:30)		Laparoscopic group (*n*:15)			*p*	
			Mean ± SD		Mean ± SD				
**Lenght of hospital stay** ^**t**^			**3.6 ± 1.58**		**1.8 ± 0.7**			**< 0.05**	
Operation time^t^ (min)			102.8 ± 22.4		93.3 ± 18.4			0.13	
**Pain score** ^**t**^			**51.1 ± 13.2**		**17.0 ± 7.2**			**< 0.05**	
**Amount of bleeding**^**z**^ (mL)			**276.6 ± 70.3**		**81.6 ± 45.7**			**< 0.05**	
		Number		%		Number	%		
Postoperative complications		2		6.6%		0	0%	0.39	
ASA	ASA 1	0		0%		3	20%	**<0.05**	
	ASA 2	10		33%		6	40%		
	ASA 3	20		66.7%		6	40%		

*p < 0,*05*, **p < 0,*01*,* SD: Standart deviation*,* t:Independent Sample T Test*,* z:Mann Whitney U Test (Mean and standard deviation values of the data)*,* mL: millilitre*,* min: minute*

## Discussion

This study highlights the comparative advantages of laparoscopic versus open repair techniques for incarcerated incisional hernias, providing significant insights into their clinical outcomes. The results demonstrate that laparoscopic repair is associated with shorter hospital stays, lower pain scores, and reduced perioperative bleeding compared to the open repair technique. These findings align with the current literature and underscore the benefits of minimally invasive surgery in emergency settings.

The significantly shorter hospital stay observed in the laparoscopic group compared to the open repair group is consistent with existing studies. For instance, Bittner et al. found that patients undergoing laparoscopic repair for ventral hernias had reduced hospitalisation times compared to those undergoing open repair [16]. This can be attributed to the smaller incisions and less extensive tissue dissection required in laparoscopic surgery, facilitating faster recovery and earlier mobilisation.

The mean EuraHS QoL pain score was significantly lower in the laparoscopic group than in the open repair group. This reduction in postoperative pain is a well-documented benefit of laparoscopic surgery. Earle et al. reported similar findings, where patients undergoing laparoscopic hernia repair experienced less postoperative pain than those undergoing open surgery [17]. The reduced pain can be explained by the minimally invasive nature of laparoscopic surgery, which causes less trauma to the abdominal wall and surrounding tissues.

The mean amount of perioperative bleeding was significantly lower in the laparoscopic group compared to the open repair group. Studies such as those by Cuccurullo et al. support this finding, demonstrating that laparoscopic techniques result in less blood loss due to better visualisation and precise dissection [18]. The reduced bleeding risk is particularly advantageous in emergency settings, where minimising blood loss is crucial for patient stability and recovery.

Although not statistically significant, the laparoscopic group had a shorter mean operative time than the open group. This trend towards shorter operative times in laparoscopic surgery is supported by studies such as those by Kokotovic et al., which suggest that the learning curve for laparoscopic techniques can eventually lead to faster surgeries as surgeons become more proficient [19].

The absence of postoperative complications in the laparoscopic group, despite a higher mean BMI compared to the open group, is noteworthy. High BMI is a known risk factor for surgical complications, as highlighted by Derici et al. [20]. However, the laparoscopic approach appears to mitigate these risks, possibly due to less extensive tissue handling and reduced wound size. This finding is consistent with the study by Silecchia et al., which reported lower complication rates in laparoscopic hernia repairs, even in obese patients [21].

The recurrence rates were similar between the two groups, with one recurrence in each group during the follow-up period. This finding suggests that both techniques effectively prevent recurrence when performed correctly. However, as noted by studies such as those by Kockerling et al., long-term follow-up is necessary to fully assess the durability of the repairs [22].

The advantages of laparoscopic repair, including shorter hospital stays, lower pain scores, and reduced perioperative bleeding, make it a preferable option for many patients. However, it is essential to consider the learning curve associated with laparoscopic techniques, which can impact operative times and outcomes. Additionally, laparoscopic surgery requires specialised equipment and training, which may not be available in all settings.

Conversely, open repair remains a viable option, particularly in cases where laparoscopic access is not feasible due to extensive adhesions or patient instability. The open technique allows for a straightforward approach to hernia reduction and repair, albeit with higher risks of postoperative pain and longer recovery times.

### Study limitations

This study has several limitations. First, the small sample size of 45 patients limits the generalizability of the findings. Larger studies are needed to validate these results. Second, the study was conducted in a single tertiary care hospital, which may introduce institutional biases and limit the applicability of the results to other settings. Additionally, the follow-up period of approximately 12 months may not capture all long-term outcomes, such as hernia recurrence and chronic pain. In addition, the non-randomized design and potential selection bias could influence the outcomes, as patients were not randomly assigned to the laparoscopic or open repair groups. Further research with larger, randomised, and multicenter trials is necessary to confirm these findings and address these limitations. Finally, the outcomes of laparoscopic surgery are highly dependent on the surgeon’s expertise and experience. The learning curve associated with laparoscopic techniques could impact the operative times and complication rates. Studies that include a standardised level of surgical proficiency or account for the learning curve are needed to provide a more accurate comparison.

## Conclusion

In conclusion, this study demonstrates that laparoscopic repair offers significant advantages over open repair for incarcerated incisional hernias, including shorter hospital stays, lower postoperative pain, and reduced perioperative bleeding. Despite the higher mean BMI in the laparoscopic group, no postoperative complications were observed, highlighting the technique’s efficacy and safety. These findings support the preferential use of laparoscopic repair in appropriate patients, potentially setting a new standard for the emergency management of incarcerated incisional hernias. Further research with larger sample sizes and longer follow-up periods is recommended to confirm these results and refine surgical guidelines.

## Electronic supplementary material

Below is the link to the electronic supplementary material.

Supplementary Material 1

## Data Availability

Data used in this study can be provided on reasonable request.
